# Rational Combination of Selenium Application Rate and Planting Density to Improve Selenium Uptake, Agronomic Traits, and Yield of Dryland Maize

**DOI:** 10.3390/plants13101327

**Published:** 2024-05-11

**Authors:** Fei Gao, Le Wang, Rong Zhao, Yixiong Wang, Yankun Ma, Rulan Yang, Qi Zhang, Chuangyun Wang

**Affiliations:** College of Agronomy, Shanxi Research Institute of Functional Agriculture, Shanxi Agricultural University, Taiyuan 030031, China; sxfeigao@sxau.edu.cn (F.G.); s20212101@stu.sxau.edu.cn (Y.W.); yankunma1218@163.com (Y.M.); z20223079@stu.sxau.edu.cn (R.Y.); z20213111@stu.sxau.edu.cn (Q.Z.)

**Keywords:** dryland maize, selenium fertilizer, sowing density, physiological characteristics, grain yield

## Abstract

Exogenous selenium application could effectively improve the selenium absorption of crops affected by different climatic conditions due to changes in the planting environment and planting conditions. We planted maize at planting densities of 67,500 plants ha^−1^ (D1) and 75,000 plants ha^−1^ (D2). Five selenium fertilizer gradients of 0 mg m^−2^ (Se0), 7.5 mg m^−2^ (Se1), 15.0 mg m^−2^ (Se2), 22.5 mg m^−2^ (Se3), and 30.0 mg m^−2^ (Se4) were applied to investigate the response of the plants to selenium fertilizer application in terms of the gradient selenium absorption and substance accumulation. With the increase in the amount of selenium fertilizer applied, more of the selenium fertilizer will be absorbed and transported from the leaves to the grains, and the selenium content of the grains will gradually increase and exceed the selenium content of leaves. Under the D2 density in 2022, the selenium content of the grains under Se1, Se2, Se3, and Se4 treatments increased by 65.67%, 72.71%, and 250.53%, respectively, compared with that of Se0. A total of 260.55% of the plants showed a gradient of grain > leaf > cob > stalk from the Se2 treatment, and the overall selenium content of the plants increased first and then decreased. Under the D1 density, compared with the Se0, the dry matter mass of the Se1, Se2, Se3, and Se4 treatments significantly improved by 5.84%, 1.49%, and 14.26% in 2021, and significantly improved by 4.84%, 3.50%, and 2.85% in 2022. The 1000-grain weight under Se2, Se3, and Se4 treatments improved by 8.57%, 9.06%, and 15.56% compared to that under the Se0 treatment, and the yield per ha under the Se2, Se3, and Se4 treatments was 18.58%, 9.09%, and 21.42% higher than that under Se0 treatment, respectively. In addition, a reasonable combination of selenium application rate and density could improve the chlorophyll content and stem growth of dryland maize. This lays a foundation for the efficient application of selenium fertilizer and provides an important reference.

## 1. Introduction 

At present, the lack of trace elements is considered to be a major global problem affecting nearly 20 million people around the world [1]. Selenium is a necessary trace mineral element, and the human body requires it to maintain certain activities [2,3]. Selenium exists in the form of selenoprotein, selenocysteine, and selenomethionine as essential micronutrients that affect important biological processes such as free radical metabolism, immune function, and apoptosis [4]. Selenium deficiency in the human body can lead to a decline in immunity and result in chronic diseases such as hypertension and diabetes [5]. 

Selenium has multiple biological functions in human and animal bodies, and its remarkable effect on plant growth and physiological development has attracted an increasing amount of attention. Studies have shown that the application of appropriate levels of selenium can boost botanical growth and development to a certain extent [6]. The rational application of selenium fertilizer has been found to not only markedly increase the selenium content of crops [7], but also promote the increase of fresh biomass of crop populations [8]. It has a certain effect on increasing the yield of rice [9] when the proper amount of selenium fertilizer is applied. Maize is widely grown all over the world and serves as a significant staple crop, so it is of great importance to improve people’s dietary selenium intake by increasing the selenium content of maize [10,11]. The application of selenium fertilizer usually varies significantly due to changes in the planting environment, which is due to the influence of different climatic conditions [12]. For example, the amount of soil moisture affects the absorption efficiency of selenium fertilizer by plants [13], light intensity affects improvements in the quality of broccoli [14], and the application of potassium humate also affects the effectiveness of selenium fertilizer [15]. In addition, selenium application that is too high or too low inhibits plant growth [6,11], while an appropriate amount of selenium promotes the growth and development of crops [16].

In addition, the application of selenium fertilizer effectively improves plants’ tolerance to drought stress [17]. Selenium improves resistance to drought by improving the physiological traits of plants [18]. For instance, the inhibitory effect of drought stress on rice growth can be alleviated by selenium fertilizer application [19].

The rapid growth of the world population has brought about food shortages, and increasing planting density to meet the demand for increasing production is one of the effective means used to address this issue [20]. Planting density is the main constraining factor for the formation of yield per unit area, and a reasonable increase in planting density is an effective measure to increase maize production [21]. The primary purpose of increasing the density is to obtain higher yields, but three factors of yield composition are mutually restricted in production practice. Excessive density will shorten the active filling period of the grain and reduce the maximum filling rate, resulting in inadequate filling and lower grain weight per ear, which will affect the yield [22]. Among the three factors of yield, the number of ears per unit area is the most easily controlled, so increasing the planting density and ensuring a sufficient number of ears of maize per unit area can effectively increase the yield. Therefore, the selection of a reasonable cultivation density is helpful to achieve the mutual balance of yield factors, enhance the utilization of light and heat resources, and improve the accumulation of benefits to achieve an increase in yield.

Under different planting density levels, dry matter accumulation undergoes regular changes. As the planting density increases, the amount of dry matter accumulation per maize plant shows a decreasing trend [23]. However, the dry matter accumulation in the maize plants as a group will exhibit a gradual increase as the planting density increases within a specific range. With a reasonable increase in the planting density, the dry matter accumulation and yield of a single plant will gradually decrease, but the population quantity advantage caused by increasing density causes the yield and dry matter accumulation of the group increase significantly. If the planting density tolerance range is exceeded, the yield per plant and the dry matter will be reduced significantly, and the population quantity advantage will not be compensated for accordingly, which will eventually lead to a decrease in the yield and dry matter accumulation of the population.

The purpose of this study was to explore the effects of different selenium application rates on the selenium content and selenium fertilizer utilization efficiency of maize in drylands with different densities and to determine the most suitable planting density and the best range of selenium fertilizer application to promote maize growth and material accumulation. This will help to provide a scientific basis for the high-yield and high-quality cultivation of maize in selenium-rich dryland.

## 2. Materials and Methods

### 2.1. Experimental Design

The experiment was conducted at the Dongyang Experimental Base of Shanxi Agricultural University (37°11′ N, 113°06′ E) from 2021 to 2022. The annual average temperature of the base was 9.80 °C, and the average annual rainfall was 422.20 mm. The soil texture type was clay. Before the planting season, topsoil was collected from each plot using a five-point sampling method, air-dried indoors, pulverized, and sieved, and the soil organic matter and major nutrient contents were determined. The nutrient contents of the soil are as follows: soil organic matter (SOM), 13.06 g kg^−1^; total nitrogen (TN), 0.75 g kg^−1^; total phosphorus (TP), 0.93 g kg^−1^; total potassium (TK), 17.08 g kg^−1^; alkaline nitrogen (AN), 66.50 mg kg^−1^; available phosphorus (AP), 10.90 mg kg^−1^; available potassium (AK), 109.00 mg kg^−1^; pH, 8.49; total selenium, 0.29 mg kg^−1^. 

The spring maize variety Dafeng 30 was selected as the experimental material, and the growth period was 127 days. The treatments consisted of two densities: 67,500 plant ha^−1^ (D1) and 75,000 plant ha^−1^ (D2); two planting densities; and five Se levels: 0 mg m^−2^ (Se0), 7.5 mg m^−2^ (Se1), 15.0 mg m^−2^ (Se2), 22.5 mg m^−2^ (Se3), and 30.0 mg m^−2^ (Se4), for a total of ten treatments. The experiment involved the replication of each treatment three times, with each plot measuring 40 m^2^ (4 m × 10 m) and being randomly arranged. A total of 30 plots were used in the study. All samples were treated with a regulated fertilizer complex (26% N:13% P:6% K) of 750 kg ha^−1^. All plots were rototilled before sowing and the composite fertilizer was applied to the farm in a single application. Selenium-rich organic fertilizer (selenium content of 1 g kg^−1^, nutrient content of NPK ≥ 5 g kg^−1^, organic matter content ≥ 45 g kg^−1^) was selected for application. The rest of the field management practices were carried out following local high-yielding field management practices, using rain-fed agricultural planting methods for management.

### 2.2. Sampling and Measurements

#### 2.2.1. Determination of Selenium Content

At the maturity stage, three maize plants with similar growth traits were randomly selected from each plot, and the selected plants were accurately categorized: stalks, leaves, kernels, and rachises, and then each part of the plant was dried in an oven at 80 °C until constant weight and weighed. In addition, the dried parts were crushed and finely ground into powder and stored in sealed paper bags.

Three randomly selected sampling sites per plot were used to collect selenium-containing soils. Soil samples were taken from the 0–20 cm, 20–40 cm, and 40–60 cm soil horizons using a soil auger. After removing debris such as stones and roots, they were sieved (2 mm), ground in the laboratory, and placed into plastic bags for subsequent determinations.

The above-obtained maize plants and soil sample powder (0.3 g) along with HNO_3_-H_2_O_2_ (*v*/*v* ratio of 3:1) were digested by a microwave digester (MARS6 One-Touch). The content of selenium in the maize was determined by hydride generation–atomic fluorescence gradient spectroscopy (AFS-975, the limit of detection was 0.01 μg L^−1^), according to the Chinese national standard GB5009.93-2017 [24] (the detection limit was 2 μg L^−1^ and the quantification limit was 6 μg L^−1^).

#### 2.2.2. Determination of Water Content in Soil

The recovered moist soil was weighed and put into an aluminum box to dry at 105 °C for 24 h. Then, the dry soil was weighed and the water content calculated.

#### 2.2.3. Determination of Soil Properties

After drying the soil, 5.0 g of each plot of collected soil was weighed and put into a 50 mL beaker. Then, 25 mL of ultra-pure water was added to the beaker, stirred, and left for 30 min. Finally, we inserted the pH electrode (PHS-3C, Shanghai, China) into the upper clarifier and recorded the data; each treatment was repeated three times. The soil chemical composition was determined indoors, the soil organic matter (SOM) was analyzed by the oxidation of organic carbon using potassium chromate (K_2_Cr_2_O_7_), and the total nitrogen (TN) was determined by the Kjeldahl method. The soil total phosphorus (TP) was determined by molybdenum–antimony inverse colorimetry, and the total potassium (TK) was determined by flame photometry. The soil AN was determined using the alkali solution diffusion method and the available phosphorus (AP) concentration was determined via the molybdenum blue method. Flame photometry was used to determine the concentration of available potassium (AK).

#### 2.2.4. Determination of Physiological Indices Related to Maize Plants

Five representative plants with similar growth were selected from each plot. Their plant height (natural vertical distance from the ground surface to the highest part of the maize leaf measured before VT, and the distance from the ground surface to the tip of the male ear of the main stem after VT) and ear height (height from the base of the cob to the ground) were measured using a ruler. A straightedge and vernier calipers were used to determine the internode lengths of the second and third nodes at the base of the sample stalks and the stalk thickness (measured on the broad side of the ellipse), respectively. Stalk strength was determined using the YYD-1 stalk strength tester (Zhejiang Top Cloud-agri Technology Co., Ltd., Hangzhou, Zhejiang, China).

LAI values were determined using the LAI-2200C Plant Canopy Analyzer (LI-COR, Lincoln, NE, USA) at each physiological period of the maize. SPAD directly reflects the relative chlorophyll content in the leaves, which is measured with a chlorophyll meter (SPAD-502 Plus, Konica Minolta Sensing, Inc., Sakai, and Osaka, Japan).

### 2.3. Statistical Analysis

All data were pre-processed using Microsoft Excel 2016 (Microsoft Corpo., Redmond, WA, USA). The treatments were compared with an analysis of variance using IBM SPSS Statistics 25.0 (SPSS Inc., Chicago, IL, USA). Multiple comparisons were made to determine the significance of the treatment effect; the least significant difference (LSD) was set to test, and the significance level was set at the probability level of 0.5. The GraphPad Prism 9.0 software (GraphPad Software, La Jolla, CA, USA) was used for plotting.

## 3. Results

### 3.1. Effects of Selenium Fertilization Amount on Selenium Content in Different Organs of Maize under Different Planting Densities 

**Figure 1:**
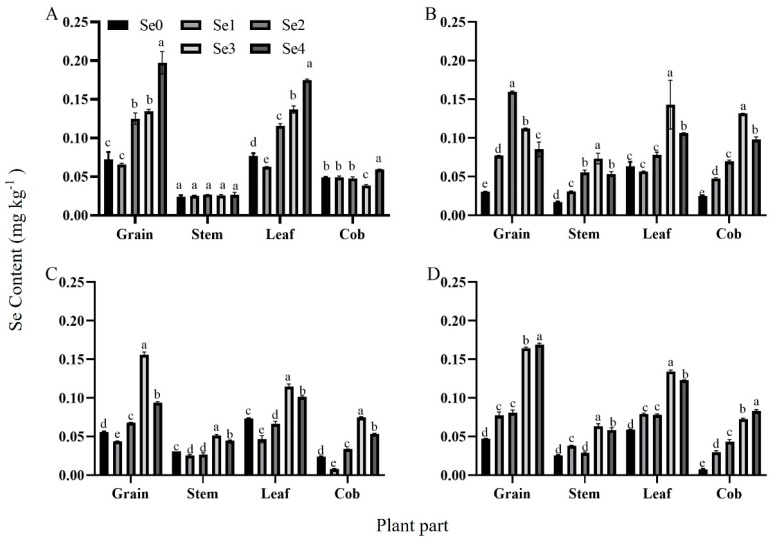
Effects of selenium application on selenium content in maize plants at different densities. Description of treatment groups: (**A**,**B**) represent 67,500 plants ha^−1^ (D1) and 75,000 plants ha^−1^ (D2), which were the two planting densities in 2021; (**C**,**D**) represent 67,500 plants ha^−1^ (D1) and 75,000 plants ha^−1^ (D2), which were the two planting densities in 2022. Se0: selenium fertilizer application of 0 g ha^−1^; Se1: selenium fertilizer application of 75 g ha^−1^; Se2: selenium fertilizer application of 150 g ha^−1^; Se3: selenium fertilizer application of 225 g ha^−1^; Se4: selenium fertilizer application of 300 g ha^−1^. Different lowercase letters in the same column indicate significant differences among different treatments (*p* < 0.05).

Different selenium fertilizer application rates showed significant differences in selenium content in the various organs of maize plants (Figure 1). In 2021, under the DI density condition, the selenium content of the grains, leaves, and cobs revealed a linear trend with the amount of selenium applied, and the maximum value was obtained under the Se4 treatment. The selenium content of the grains, leaves, and cobs increased by 173.27%, 127.05%, and 219.26%, respectively, compared with Se0, which had a significant improvement effect; under the D2 density condition, the selenium content of the stems, grains, leaves, and cobs of the maize increased in the early stage and decreased later upon the application of selenium fertilizer. The selenium content of the grain reached the maximum under the Se2 treatment. Under the Se3 treatment, the selenium content of the stems, leaves, and cobs reached the maximum, which was 329.24%, 125.20%, and 425.50% higher than that of the Se0 treatment, respectively. In 2022, at the D1 density, the selenium content of each organ of the plant increased first and then decreased upon the implementation of the increase in the selenium content. The maximum value was obtained under the Se3 treatment, which was 179.93%, 66.78%, 56.40%, and 212.97% higher than the Se0 treatment. Under the D2 density condition, the selenium content of grains and cobs increased with the increase in the selenium dosage. The selenium content of grains in the Se1, Se2, Se3, and Se4 treatments was 65.67%, 72.71%, 250.53%, and 260.55% higher than in those treated with Se0, respectively. From the beginning of the Se2 treatment, the selenium content of the grains exceeded the leaves. With the increase in the selenium content, the selenium content in stems and leaves increased and then decreased, and reached the maximum under the Se3 treatment. In the two-year experiment, the maximum selenium content in each treatment was found in the grains.

### 3.2. Effects of Selenium Fertilization Amount on Selenium Content in Different Soil Layers under Different Densities

**Figure 2:**
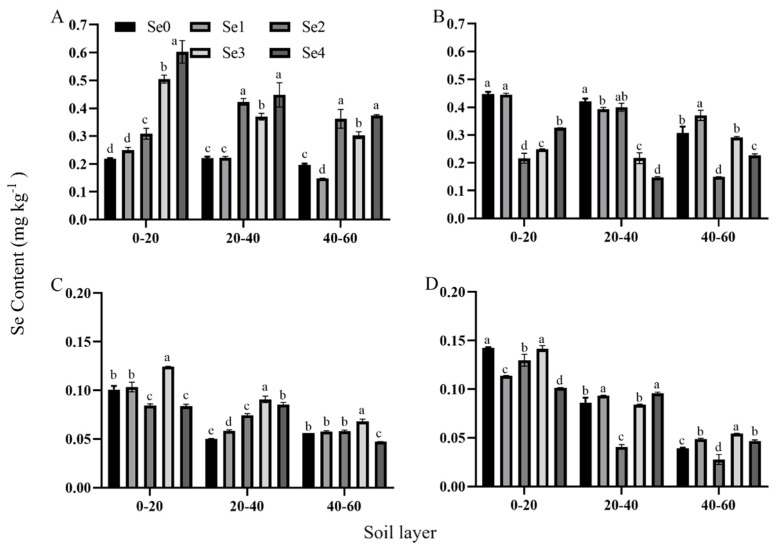
Effects of selenium application amount on selenium content in soil layers with different densities. Description of treatment groups: (**A**,**B**) represent 67,500 plants ha^−1^ (D1) and 75,000 plants ha^−1^ (D2), which were the two planting densities in 2021; (**C**,**D**) represent 67,500 plants ha^−1^ (D1) and 75,000 plants ha^−1^ (D2), which were the two planting densities in 2022. Se0: selenium fertilizer application of 0 g ha^−1^; Se1: selenium fertilizer application of 75 g ha^−1^; Se2: selenium fertilizer application of 150 g ha^−1^; Se3: selenium fertilizer application of 225 g ha^−1^; Se4: selenium fertilizer application of 300 g ha^−1^. Different lowercase letters in the same column indicate significant differences among different treatments (*p* < 0.05).

When the selenium fertilizer gradient treatment was carried out under different planting densities, the selenium content in each soil layer was significantly different (Figure 2). In 2021, under the D1 density condition, the selenium content in the different soil thicknesses showed a curve change with the increasing amount of selenium fertilizer applied. The selenium content at soil thicknesses of 0–20 cm, 20–40 cm, and 40–60 cm increased with the increase in the amount of selenium applied. The maximum value was obtained under the Se4 treatment, which was 176.28%, 102.71%, and 91.30% higher than for Se0, respectively. Under the other treatments, the selenium fertilizer application led to no significant differences in the selenium content in the soil under the conditions of different planting densities.

### 3.3. Effects of Selenium Fertilization Amount on Soil pH Value and Water Content under Different Densities

The variety of planting environments affects the effectiveness of exogenous selenium fertilizer. Under the D1 density condition in 2021, the pH value in the soil gradually decreased with the application of selenium fertilizer (Table 1). Under the Se3 treatment, the soil layers at depths of 0–20 cm, 20–40 cm, and 40–60 cm reduced to 2.23%, 3.53%, and 3.54% compared with the Se0 treatment, but the difference was not significant under the D2 density. Under the D1 density condition in 2021, the soil water content exhibited a trend of increasing upon the application of selenium fertilizer (Table 2). Under the Se3 treatment, the soil layers at 0–20 cm, 20–40 cm, and 40–60 cm increased by 53.85%, 20.00%, and 15.38%, respectively, compared with Se0 treatment. Under the Se4 treatment, the soil layers at 0–20 cm, 20–40 cm, and 40–60 cm decreased by 46.16%, 33.33%, and 7.69%, respectively, compared with the Se0 treatment, but the soil water content increased under the D2 density. Soil water content and soil pH value have a significant effect on the effective absorption of selenium, which has reference significance for judging the absorption of selenium.

### 3.4. Effect of Selenium Fertilization Amount on Dry Matter Accumulation and Distribution of Maize under Different Densities

The gradient application of the selenium fertilizer made the dry matter accumulation of the plants under different cultivation densities significantly different (Table 3). Under the same planting density, the two-year test results of selenium fertilizer treatment were similar. Under the D1 density condition, the gradient application of the selenium fertilizer made the dry matter weight of the ears and total plant increase first and then decrease, and all reached the maximum value under the Se3 treatment. In 2021, they increased by 7.33% and 14.26%, respectively, and in 2022, they increased by 8.34% and 2.85% compared with the Se0 treatment. Under the D2 density condition, the dry matter accumulation of maize also first increased, followed by a subsequent decrease with the gradient application of the selenium fertilizer. The maize ear dry matter mass and total plant dry matter mass reached the maximum under the Se1 treatment, which was 12.35% and 8.24% higher than that of Se0.

### 3.5. Effect of Selenium Fertilization Amount on Maize Yield under Different Densities

The gradient utilization of selenium fertilizer caused significant differences in the yield (Table 4). Under the D1 density, the utilization of selenium fertilizer resulted in a significant difference in the 1000-grain weight, which led to a significant difference in yield. The 1000-grain weight increased by 8.57%, 9.06%, and 15.56% under the Se2, Se3, and Se4 treatments compared with Se0, respectively. The grain yield per hectare increased by 18.58%, 9.09%, and 21.42% under the Se2, Se3, and Se4 treatments compared with Se0, respectively. At the D2 density, the application of selenium fertilizer had no significant effect on yield.

### 3.6. Effects of Selenium Fertilization Amount on Leaf Area and Chlorophyll Content under Different Densities

The change in the selenium application rate changed the chlorophyll content and leaf area of the maize under different planting densities (Table 5). Under the low-density condition, the chlorophyll content of the leaves gradually increased with the growth process. Under the D1 density, the chlorophyll content under the Se4 treatment increased by 1.02%, 2.39%, and 8.29%, respectively, compared with that of Se0 in 2021. With the increase in the density, it showed a decreasing trend. Under the D2 density, the chlorophyll content under the Se2 treatment increased by 3.11%, 0.70%, 47.69%, and 72.84%, respectively, compared with the Se0 treatment during the period of V6, VT, and R3 in 2021. Compared with the Se0 treatment, the chlorophyll content increased by 2.49%, 0.05%, and 72.84%, respectively. In 2022, the chlorophyll content of the Se2-treated leaves increased by 6.30%, 3.62%, and 0.83%, respectively, compared with the Se0 treatment. Under the same density conditions, the chlorophyll content of the leaves increased at first and then decreased with the increase in selenium application. Under the D2 density, in the V6 period of 2021, the chlorophyll content of leaves under the Se1, Se2, Se3, and Se4 treatments increased by 0.82%, 3.11%, 2.49%, and 0.20%, respectively, compared with the Se0 treatment. In the V6 period of 2022, the chlorophyll content of the leaves grown under the Se1, Se2, Se3, and Se4 treatments increased by 2.20%, 6.30%, 1.30%, and 1.76%, respectively, compared with the Se0 treatment. 

The leaf area decreased gradually with the growth process. For example, in 2021, during the VT, R3 period, the D1 planting density of the Se4 treatment increased by 2.51% and 0.11%, respectively, compared with that of the Se0 treatment. The planting density of the Se4 treatment increased by 33.29% and 3.81%, respectively, compared with the Se0 treatment during the VT, R3 period. Under the D2 planting density in 2022, the Se4 treatment increased by 0.21% and 1.36% compared with the Se0 treatment during the VT, R3 period. The application of selenium fertilizer increased the leaf area of maize grown under the single planting density. For example, during the VT period under the D2 planting density in 2021, the leaf area increased by 13.92% under the Se1, Se2, Se3, and Se4 treatments compared with the Se0 treatment, respectively. However, under the D2 planting density in 2022, the leaf area in the R3 period showed the opposite trend. Compared with the Se0 treatment, the leaf area under the Se4 treatment decreased by 1.21%, 15.28%, 12.58%, and 1.36%, respectively. Thus, it can be seen that the application of an appropriate amount of selenium is beneficial as it increases the chlorophyll content and the leaf area.

### 3.7. Effects of Selenium Fertilization Amount on Plant Height and Ear Height under Different Densities of Dryland Maize

The change in the selenium application rate changed the plant height and ear height of the maize under different planting densities (Table 6). The inhibitory effect of selenium fertilizer application on maize plant height under single planting density gradually increased with the advance of the growth process. Under the D2 planting density in 2021, VT and R3 decreased by 2.04% and 2.70%, respectively, under Se2 treatment compared with the Se0 treatment. In the VT period, the inhibitory effect of selenium application on plant height gradually increased with the increase in the planting density: under the planting density of D1 and D2 in 2022, the inhibition effect of selenium application on plant height in the VT period decreased by 0.85% and 4.19%, respectively, compared with the Se0 treatment and the Se4 treatment. In the R3 period, the inhibitory effect of selenium application on plant height decreased gradually with the increase in the planting density: under the D1 and D2 planting density in 2021, R3 decreased by 3.00% and 2.38%, respectively, compared with Se0 treatment in 2021; under the D1 and D2 planting density in 2022, R3 decreased by 0.42% and 0.24%, respectively, compared with the Se0 treatment in 2022. 

Under the low-density condition, the inhibitory effect of the selenium fertilizer on ear height gradually increased with the advance of the maize growth process. Under the D1 density in 2021, the Se1, Se2, and Se3 treatments in the VT period decreased by −1.72%, compared with the Se0, by −1.72% and 0.15%, respectively. The Se1, Se2, Se3, and Se4 treatments in the R3 period decreased by 14.62%, 13.75%, 5.85%, and 5.55%, respectively, compared with Se0. The inhibition effect of the R3 period was better than that of the VT period, and the same result was obtained in the 2022 planting season. In the VT period, with the increase in the planting density, the inhibitory effect of the selenium fertilizer on ear height showed an increasing trend: for example, under the D1 and D3 planting densities in 2022, Se3 treatment decreased the ear height by 0.71% and 4.74%, respectively, compared with the Se0 treatment. In the R3 period, with the increase in the planting density, the inhibitory effect of the selenium fertilizer on ear height showed a weakening trend: for example, under the D1 and D3 planting densities in 2021, Se3 treatment decreased the ear height by 5.85% compared with the Se0 treatment. The plant height and ear height of the maize had a certain response to the application of the selenium fertilizer, and the effect of the selenium fertilizer changed with the growth process.

### 3.8. Effect of Selenium Fertilization Amount on Internodal Length under Different Densities of Dryland Maize

The change in the selenium application rate was effective for the stem internode length for the maize at each growth stage under different planting densities (Table 7). For the same density, the inhibitory effect of the selenium fertilizer on the internode length increased gradually with the growth process. Under the D2 density in 2021, the Se1, Se2, Se3, and Se4 treatments of the fifth stem node in the VT period decreased by 0.82% and 3.35%, respectively, compared with Se0. The Se1, Se2, Se3, and Se4 treatments of the fifth stem node in the R3 period decreased by 7.25%, 16.07%, and 19.18% compared with Se0. With the increase in density, the inhibitory effect of the selenium fertilizer on the internode length gradually weakened. During the R3 period in 2022, the Se2 and Se3 treatments of the fourth stem node under the D1 density decreased by 5.62% compared with Se0, respectively. Under the D2 density, the Se2 and Se3 treatments of the fourth stem node decreased by 1.26% and 8.69%, respectively, compared with Se0. With the increase in the maize planting density, the application efficiency of the selenium fertilizer decreased gradually, and it also changed with the change in the growth period.

### 3.9. Effect of Selenium Fertilization Amount on Internodal Diameter under Different Densities of Dryland Maize

The change in the selenium application rate had a significant effect on the stem internode diameter of the maize at each growth stage under different planting densities (Table 8). Under the same density conditions, the inhibitory effect of the selenium fertilizer on the internode length gradually weakened with the growth process: under the D2 density in 2022, the Se3 and Se4 treatments of the fifth stem node in the VT period decreased by 7.10% and 1.82%, respectively, compared with Se0, and the Se3 and Se4 treatments of the fifth stem node in the R3 period decreased by 3.66% and 2.44% compared with Se0, showing a gradually weakening trend. For the maize stalk diameter under the single planting density, the effect of the selenium fertilizer increased first and then decreased: for example, under the D2 planting density in 2022, the Se1, Se2, and Se3 treatments of the third stem node in the R3 period increased by 2.38% and 2.25%, respectively, compared with Se0 at 1.27%. Under the D1 planting density in 2022, the Se2 and Se3 treatments of the fourth stem node in the R3 period increased by 0.66% and 5.09%, respectively. In addition, with the increase in the planting density, the inhibitory effect of the selenium fertilizer on the internode length gradually weakened: for the fourth stem node of the maize during the VT period in 2022, under the D1 planting density, the Se1 treatment decreased the internode length by 8.01% compared to Se0, and under the D2 planting density, the Se1 treatment decreased it by 3.22%. For the fourth stem node of the maize in the R3 period in 2022, the Se1 treatment decreased the internode length by 5.86% compared with Se0 under the D1 planting density, and the Se1 treatment decreased by −1.26% compared with Se0 under the D1 planting density, showing a gradually decreasing trend.

### 3.10. Effect of Selenium Fertilization Amount on Stem Strength under Different Densities of Dryland Maize

The change in the selenium application rate had an effect on the stem strength of the maize at each growth stage under different planting densities (Table 9). The application of selenium fertilizer was found to weaken the stalk strength of the maize with the advance in the growth process: under the D1 density condition in 2022, the Se3 and Se4 treatments of the fifth stem node in the VT stage increased by 45.46% and 17.13%, respectively, while in the R3 stage, the Se3 and Se4 treatments of the fifth stem node decreased by 1.25% and 10.06% compared with Se0. Under the D1 density condition in 2022, the Se3 and Se4 treatments of the third stem node in the VT period increased the stalk strength by 9.18% and 4.27%, respectively, compared with Se0, while in the R3 period, the Se3 and Se4 treatments of the third stem node decreased it by 11.87%, 11.22%, 4.98%, and 7.30%, respectively, compared with Se0. Under the same density conditions, the effect of selenium fertilizer on stem strength showed a downward trend: for example, under the D2 density condition in 2022, the Se1, Se2, and Se3 treatments of the third stem node in the VT period increased the stalk strength by 4.20%, 5.42%, and 1.30%, respectively, compared with Se0. In addition, the stalk strength of the maize gradually decreased with the increase in the planting density: during the VT period in 2021, the Se3 and Se4 treatments of the third stem node under the D1 density increased the stalk strength by 9.18% compared with Se0, respectively. At the D2 density, the Se3 and Se4 treatment of the third stem node increased the stalk strength by 1.30% and 1.95% compared with Se0.

## 4. Discussion 

### 4.1. Effects of selenium Fertilization on Selenium Content under Different Fertilization Densities

**Figure 3:**
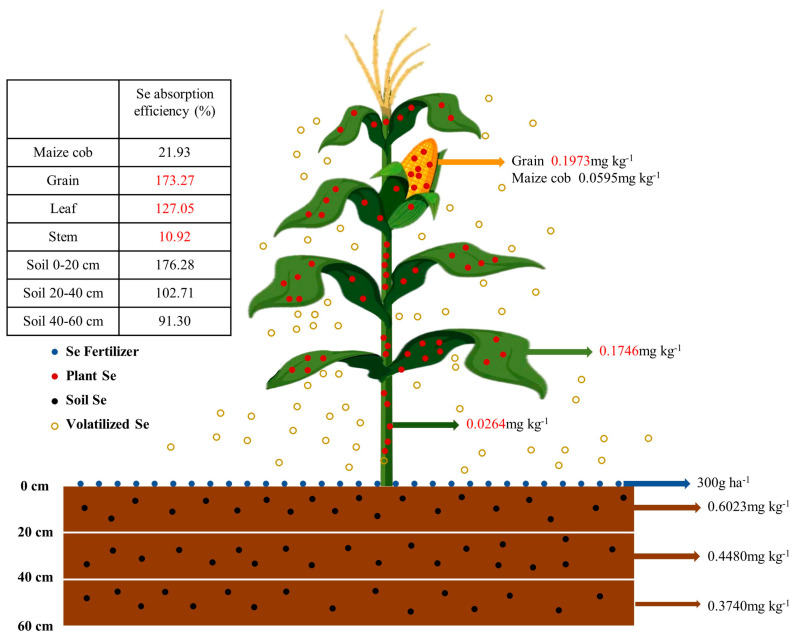
Schematic diagram of exogenous selenium absorption by maize under Se4 treatment. The red numbers on the left are the absorption efficiencies of maize stem, leaf and grain in the Se4 treatment compared to the Se0 treatment, and the red numbers on the right are the selenium content of each part of the maize plant.

Applying exogenous selenium fertilizer to crops is an effective way to increase the selenium content in the edible portion of crops, and solve the problem of selenium deficiency in humans in selenium-deficient areas [25]. In this study, it was found that the selenium content of the plants showed a linear increase with the increase in the amount of selenium fertilizer applied. Studies have shown that the absorption of selenium by plants is influenced by many factors, such as the application method, type, and amount [26]. Previous studies have shown that the application of selenium fertilizer can effectively increase the selenium content in plants [27]. In the different crops, the main enrichment sites of selenium were different. Under the two planting density conditions in this study (Figure 1), the Se0 and Se1 treatments mainly showed a gradient of selenium content in leaf > grain > cob > stem; this is consistent with the conclusion of Wang et al. [28]. Under the Se2, Se3, and Se4 treatments (Figure 3), the main selenium content gradient was grain > leaf > cob > stem, which was the same as the findings of Li et al. [29]. Chen et al. found that during the vegetative growth stage of crops, the selenium absorbed by the roots was mainly distributed in the leaves after being transferred through the stems, while in the reproductive growth stage, the grains had the strongest enrichment ability for selenium [30]. This study verified that exogenous selenium was transported to the grains through the leaves as the plants grew; the selenium content in the grains gradually augmented with the increase in the application of the exogenous selenium fertilizer and, finally, contained the highest selenium content. In addition, selenium is usually absorbed and transferred in plants by the roots or leaves and transported to various organs through plasmodesma or transporters [31]. In this study, exogenous selenium was supplemented by the basal application of selenium fertilizer. The plant absorbed the selenium in the soil and then transported it through the stem. Previous studies have confirmed that the soil water content affects the absorption of selenium fertilizer by plants; metals and organics will adsorb to form complex compounds with pH changes, ultimately changing the content of soluble selenium [32]. The soluble selenium content in the soil was the main form absorbed by the maize, so soil pH was the main factor affecting the biological availability of the selenium in the soil [33]. Therefore, the plant selenium content and soil selenium content under the D1 planting density in 2021 were higher than those under the D2 planting density, but in 2022, the opposite was found. The basic application of selenium fertilizer has important reference significance for the efficient production of selenium-rich agricultural products by adjusting the soil pH and improving the utilization rate of exogenous selenium fertilizer. 

### 4.2. Effect of Selenium Fertilization on Maize Yield under Different Densities

Maize yield is mainly composed of the number of ears per unit area, the number of grains per ear, and the grain weight. The planting density of maize is one of the key factors affecting maize yield. Maize yield exhibits an initial increase followed by a subsequent decrease with the density. That is, within the appropriate planting density range, an increase in the density can increase the maize yield, but when the planting density is too high, the number of grains and the grain weight will decrease, and the maize yield will gradually decline [34]. The proper application of selenium fertilizer can effectively and slowly relieve the negative impact of increasing density on yield. Numerous academic studies have demonstrated that the utilization of selenium fertilizer yields a significant enhancement in crop productivity, particularly in maize [35] and wheat [36]. We also confirmed that applying selenium fertilizer had a certain significant effect on increasing the yield of dryland maize. In 2021, under the D1 density condition, the yield of maize significantly increased by 18.58%, 9.09%, and 21.43% in Se2, Se3, and Se4 treatments compared with the Se0 treatment. In this study, the effect of selenium fertilizer application on maize yield under high-density planting conditions was not obvious. The main reason was that the density increased the competition among individual plants for resources, which limited the maize yield potential, resulting in a decrease in yield as the effect of the selenium fertilizer was limited. Thus, it is essential to enhance the quality and yield of crops in an appropriate way to cope with the increasing demand for food [37]. We verified the gradient application of selenium fertilizer and confirmed that the maize yield trend increased and then decreased due to the effect of selenium fertilizer; this is consistent with the conclusion of Chernikova et al. [8], who reported that low-dose selenium fertilizer promotes crops and high-dose selenium fertilizer produces toxic effects. Thus, adding selenium fertilizer into the field can improve the material accumulation and yield of crops, which has significance for the guidance of agricultural production. 

### 4.3. Effect of Selenium Fertilization on Maize Physiological Characteristics under Different Densities

The results showed that the leaf area and chlorophyll content of the maize decreased with the increase in the planting density [38,39]. In this study, leaf area and chlorophyll content showed a downward trend with the change in density. Selenium can regulate the biosynthesis of porphyrin in plants. Because the formation of chlorophyll is related to porphyrin, the addition of selenium fertilizer can promote the formation of chlorophyll [40]. Duan et al. [41] showed that the application of selenium could increase the content of chlorophyll in leaves, slow down leaf senescence, and be beneficial to sustainable growth. The study by Gong Rui [42] showed that with the increase in selenium application, the chlorophyll content of the leaves increased at first and then decreased. A high selenium concentration will reduce the chlorophyll content of the leaves, resulting in the accelerated senescence of waxy maize leaves. This study showed that the inhibition of leaf area and chlorophyll content caused by the increase in density could be weakened with an increase in selenium application. 

The results showed that plant height and ear height decreased with the increase in density, which was consistent with the results of many studies [43,44,45]. With the change in plant height and ear height, the internode length and internode diameter of the plant will also change, which will affect the lodging resistance of the plant. Therefore, stem strength is closely related to planting density, and the lodging resistance of the maize decreases gradually with the increase in planting density [46]. In this study, the selenium application inhibited plant height and ear height in varying degrees, which was contrary to the results of Chernikov [47] and other studies on maize, which was mainly due to the effects of the planting varieties and cultivation environment. In this study, the application of the selenium fertilizer promoted the stem diameter in varying degrees, but the promoting effect gradually weakened with the advance in the growth period. Luo et al. [48] confirmed that the application of selenium can significantly increase the stem diameter of rice under drought stress. The application of exogenous selenium could improve the anatomical characteristics of wheat stems and increase the stem diameter of wheat [49]. The lodging resistance of maize stalk is very important in the process of maize growth. Stalk lodging in different periods will seriously affect the growth, yield, quality, and mechanized harvest of maize. The application of an appropriate amount of selenium fertilizer to increase the stalk strength of maize plants and reduce the lodging in planting has important practical significance for the cultivation of upland maize.

## 5. Conclusions

The results showed that the proper application of selenium fertilizer could improve the yield of maize. Combined with the planting situation over two years, the best yield was 12,519.0–16,242.2 kg ha^−1^, when the planting density was 67,500 plants ha^−1^ and the selenium application rate was 150 g ha^−1^. In addition, with the increase in selenium fertilizer application, the transfer rate of selenium from leaves to grains can be increased, which is of great significance for selenium-rich crop production. The difference in planting environment affects the efficiency of exogenous selenium. Adjusting the soil pH and water content maximizes the absorption efficiency of selenium fertilizer and realizes the efficient production of selenium-rich agricultural products.

## Figures and Tables

**Table 1:** Effects of gradient application of selenium fertilizer on pH of different soil layers in 2021 and 2022.

Year	Density	Treatment	Soil Layer Thickness		
			0–20 cm	20–40 cm	40–60 cm
2021	D1	Se0	7.62 a	7.64 a	7.63 a
		Se1	7.67 a	7.59 a	7.60 a
		Se2	7.57 a	7.51 a	7.38 b
		Se3	7.45 b	7.37 a	7.36 b
		Se4	7.37 b	7.50 a	7.48 b
	D2	Se0	7.57 a	7.56 ab	7.54 c
		Se1	7.60 a	7.67 a	7.75 a
		Se2	7.54 a	7.64 a	7.60 bc
		Se3	7.62 a	7.56 ab	7.60 b
		Se4	7.56 a	7.49 b	7.48 d
2022	D1	Se0	7.81 c	8.09 ab	8.13 b
		Se1	7.95 b	8.06 b	8.11 b
		Se2	8.03 a	8.10 ab	8.19 a
		Se3	8.06 a	8.09 ab	8.20 a
		Se4	8.04 a	8.13 a	8.20 a
	D2	Se0	7.68 b	7.98 ab	8.02 c
		Se1	7.87 a	7.93 b	8.03 c
		Se2	7.88 a	8.05 ab	8.12 b
		Se3	7.94 a	8.06 ab	8.15 a
		Se4	7.88 a	8.11 a	8.14 ab
Y			**	**	**
D			*	NS	NS
T			**	NS	**
Y×D			**	*	**
Y×T			**	**	**
D×T			*	NS	**
Y×D×T			**	NS	**

Description of treatment groups: Se0: Se fertilizer application of 0 g ha^−1^; Se1: Se fertilizer application of 75 g ha^−1^; Se2: Se fertilizer application of 150 g ha^−1^; Se3: Se application of 225 g ha^−1^; Se4: Se fertilizer application of 300 g ha^−1^. Different lowercase letters in the same column indicate significant differences among different treatments (*p* < 0.05). NS: not significant. * Significant at the 0.05 probability level. ** Significant at the 0.01 probability level.

**Table 2:** Effects of gradient application of selenium fertilizer on the water content of different soil layers in 2021 and 2022.

Year	Density	Treatment	Soil Layer Thickness		
			0–20 cm	20–40 cm	40–60 cm
2021	D1	Se0	0.14 b	0.14 c	0.13 b
		Se1	0.17 ab	0.17 abc	0.14 ab
		Se2	0.16 ab	0.16 bc	0.14 ab
		Se3	0.18 a	0.19 a	0.15 a
		Se4	0.19 a	0.18 ab	0.15 ab
	D2	Se0	0.16 a	0.18 a	0.16 ab
		Se1	0.15 a	0.18 ab	0.13 c
		Se2	0.15 a	0.15 c	0.15 b
		Se3	0.14 a	0.15 c	0.13 c
		Se4	0.15 a	0.16 bc	0.17 a
2022	D1	Se0	0.14 a	0.19 a	0.23 a
		Se1	0.13 a	0.19 a	0.17 b
		Se2	0.15 a	0.17 a	0.23 a
		Se3	0.17 a	0.18 a	0.13 c
		Se4	0.15 a	0.19 a	0.18 b
	D2	Se0	0.17 a	0.16 a	0.18 a
		Se1	0.20 a	0.20 a	0.17 a
		Se2	0.16 a	0.19 a	0.16 a
		Se3	0.16 a	0.17 a	0.19 a
		Se4	0.17 a	0.20 a	0.17 a
Y			NS	**	**
D			NS	NS	NS
T			**	**	**
Y×D			**	NS	*
Y×T			**	**	**
D×T			**	*	**
Y×D×T			**	*	**

Description of treatment groups: Se0: Se fertilizer application of 0 g ha^−1^; Se1: Se fertilizer application of 75 g ha^−1^; Se2: Se fertilizer application of 150 g ha^−1^; Se3: Se application of 225 g ha^−1^; Se4: Se fertilizer application of 300 g ha^−1^. Different lowercase letters in the same column indicate significant differences among different treatments (*p* < 0.05). NS: not significant. * Significant at the 0.05 probability level. ** Significant at the 0.01 probability level.

**Table 3:** Effect of gradient application of selenium fertilizer on dry matter quality of dryland maize in 2021 and 2022.

Year	Density	Treatment	Stem (g)	Leaf (g)	Ear (g)	Grain (g)	Maize Cob (g)	Total Weight (g)
2021	D1	Se0	83.91 e	41.14 b	248.21 a	26.56 a	220.44 a	373.25 b
		Se1	114.56 b	35.56 c	189.11 c	25.40 a	165.77 c	339.22 c
		Se2	105.74 c	37.66 c	185.68 c	27.70 a	160.69 c	329.07 c
		Se3	128.91 a	44.37 a	253.19 a	28.11 a	223.73 a	426.47 a
		Se4	97.01 d	31.72 d	215.36 b	26.56 a	190.26 b	344.08 c
	D2	Se0	84.84 b	28.44 b	188.17 b	23.82 a	164.99 c	301.14 bc
		Se1	96.95 a	35.90 a	231.22 a	25.02 a	205.44 a	364.06 a
		Se2	87.69 b	28.24 b	199.25 b	22.63 ab	176.03 b	315.18 b
		Se3	59.99 c	22.07 c	140.21 d	18.63 b	123.52 d	222.27 d
		Se4	94.19 a	22.48 c	170.71 c	20.88 ab	157.30 c	287.37 c
2022	D1	Se0	68.33 b	73.59 a	221.06 ab	193.65 bc	27.41 a	362.98 a
		Se1	76.62 a	70.74 a	215.43 bc	187.84 cd	27.59 a	362.79 a
		Se2	76.16 a	68.53 a	208.42 c	183.61 d	24.81 a	353.11 a
		Se3	71.44 ab	70.50 a	231.39 a	203.03 a	28.37 a	373.33 a
		Se4	68.30 b	76.99 a	224.50 ab	197.84 ab	26.67 a	369.79 a
	D2	Se0	68.26 c	72.44 a	218.39 b	191.92 b	26.47 b	359.08 b
		Se1	76.57 ab	66.74 a	245.37 a	215.62 a	29.75 a	388.67 a
		Se2	72.69 bc	71.93 a	239.48 a	209.54 a	29.95 a	384.10 ab
		Se3	55.68 d	69.90 a	190.39 c	166.26 c	24.13 b	315.97 c
		Se4	80.79 a	56.15 b	129.57 d	112.16 d	17.41 c	266.51 d
Y			**	**	**	**	**	**
D			**	**	**	**	**	**
T			**	**	**	**	**	**
Y×D			**	**	**	**	**	**
Y×T			**	NS	**	**	**	**
D×T			**	**	**	**	**	**
Y×D×T			**	**	**	**	**	**

Description of treatment groups: Se0: Se fertilizer application of 0 g ha^−1^; Se1: Se fertilizer application of 75 g ha^−1^; Se2: Se fertilizer application of 150 g ha^−1^; Se3: Se fertilizer application of 225 g ha^−1^; Se4: Se fertilizer application of 300 g ha^−1^. Different lowercase letters in the same column indicate significant differences among different treatments (*p* < 0.05). NS: not significant. ** Significant at the 0.01 probability level.

**Table 4:** Effect of gradient application of selenium fertilizer on yield of dryland maize in 2021 and 2022.

Year	Density	Treatment	Number of Rows per Ear	Row Number	1000-Grain Weight (g)	Grain Yield per Hectare (kg)
2021	D1	Se0	16.63 b	36.81 a	317.19 b	13,697.69 bc
		Se1	17.16 ab	35.38 a	303.13 b	13,334.92 c
		Se2	17.31 b	36.69 a	344.38 a	16,242.15 a
		Se3	17.44 b	35.03 a	345.94 a	14,942.98 b
		Se4	16.94 ab	34.50 a	366.56 a	16,632.38 a
	D2	Se0	17 a	38.31 b	320.00 a	14,704.36 a
		Se1	17.63 a	36.91 b	290.63 bc	13,228.17 b
		Se2	17.06 a	41.19 a	305.31 ab	14,698.13 a
		Se3	17 a	37.94 b	287.50 c	13,173.98 b
		Se4	17 a	39.16 ab	316.56 a	13,911.84 ab
2022	D1	Se0	16.03 a	38.56 b	316.94 a	13,198.95 ab
		Se1	16.91 ab	36.18 a	313.53 a	12,919.06 a
		Se2	17.13 a	38.31 b	321.82 a	14,206.72 b
		Se3	16.79 ab	37.69 ab	308.25 a	13,118.14 ab
		Se4	16.42 ab	38.91 b	321.31 a	13,838.54 ab
	D2	Se0	17.16 b	37.78 a	310.93 a	15,066.56 b
		Se1	16.75 b	37.88 a	309.23 a	14,686.43 b
		Se2	16.82 b	37.31 a	321.89 a	15,116.06 b
		Se3	15.68 a	36.99 a	315.68 a	13,676.79 ab
		Se4	15.78 a	36.44 a	300.84 a	12,973.98 a
Y			**	NS	NS	*
D			NS	**	**	NS
T			*	NS	*	**
Y×D			NS	**	**	**
Y×T			NS	NS	*	NS
D×T			**	NS	NS	**
Y×D×T			NS	NS	NS	NS

Description of treatment groups: Se0: Se fertilizer application of 0 g ha^−1^; Se1: Se fertilizer application of 75 g ha^−1^; Se2: Se fertilizer application of 150 g ha^−1^; Se3: Se fertilizer application of 225 g ha^−1^; Se4: Se fertilizer application of 300 g ha^−1^. Different lowercase letters in the same column indicate significant differences among different treatments (*p* < 0.05). NS: not significant. * Significant at the 0.05 probability level. ** Significant at the 0.01 probability level.

**Table 5:** Effect of gradient application of selenium fertilizer on leaf area and chlorophyll content of dryland maize in 2021 and 2022.

Year	Density	Treatment	Chlorophyll Content (SPAD Plant^−1^)			Leaf Area (cm^2^ Plant^−1^)	
			Bighorn Mouth Stage	Tasseling	Milk Stage	Tasseling	Milk Stage
2021	D1	Se0	58.72 a	62.00 a	49.47 ab	499.09 a	711.65 a
		Se1	58.00 a	60.33 a	43.33 b	527.33 a	719.83 a
		Se2	56.70 a	61.38 a	48.23 ab	473.36 a	699.27 a
		Se3	55.57 a	62.00 a	56.43 a	536.43 a	685.08 a
		Se4	58.12 a	63.48 a	53.57 ab	511.62 a	710.89 a
	D2	Se0	56.27 a	60.08 a	48.23 a	457.19 a	659.66 a
		Se1	56.73 a	58.85 a	41.37 a	520.81 a	734.64 a
		Se2	58.02 a	60.50 a	25.23 b	604.93 a	693.20 a
		Se3	57.67 a	60.05 a	13.10 c	608.82 a	675.42 a
		Se4	56.38 a	59.55 a	12.73 c	609.39 a	684.76 a
2022	D1	Se0	57.52 a	56.12 a	60.78 a	645.17 a	776.52 a
		Se1	55.80 a	58.60 a	61.70 a	637.77 a	756.05 a
		Se2	56.83 a	56.03 a	60.57 a	633.72 a	720.03 a
		Se3	56.95 a	58.58 a	61.10 a	616.17 a	747.55 a
		Se4	55.82 a	59.30 a	58.70 a	598.03 a	768.80 a
	D2	Se0	55.53 ab	57.52 ab	60.18 a	610.20 a	775.60 a
		Se1	56.75 ab	58.00 a	58.35 a	623.83 a	766.25 a
		Se2	59.03 a	59.60 a	60.68 a	603.70 a	657.10 a
		Se3	56.25 ab	57.10 b	57.98 a	621.28 a	678.02 a
		Se4	54.55 b	59.05 ab	58.45 a	611.50 a	765.08 a
Y			NS	**	**	**	**
D			NS	NS	**	NS	NS
T			NS	NS	**	NS	NS
Y×D			NS	*	**	NS	NS
Y×T			NS	NS	**	NS	NS
D×T			*	NS	**	NS	NS
Y×D×T			NS	NS	**	NS	NS

Description of treatment groups: Se0: Se fertilizer application of 0 g ha^−1^; Se1: Se fertilizer application of 75 g ha^−1^; Se2: Se fertilizer application of 150 g ha^−1^; Se3: Se fertilizer application of 225 g ha^−1^; Se4: Se fertilizer application of 300 g ha^−1^. Different lowercase letters in the same column indicate significant differences among different treatments (*p* < 0.05). NS: not significant. * Significant at the 0.05 probability level. ** Significant at the 0.01 probability level.

**Table 6:** Effect of gradient application of selenium fertilizer on plant height and ear height of dryland maize in 2021 and 2022.

Year	Density	Treatment	Plant Height (cm)		Ear Height (cm)	
			Tasseling	Milk Stage	Tasseling	Milk Stage
2021	D1	Se0	303.67 a	312.00 a	106.17 a	114.00 a
		Se1	307.00 a	305.67 ab	108.00 a	97.33 b
		Se2	305.33 a	293.00 b	106.33 a	98.33 b
		Se3	306.17 a	302.67 ab	110.00 a	107.33 ab
		Se4	304.17 a	314.67 a	105.33 a	107.67 ab
	D2	Se0	310.33 a	308.00 a	114.33 a	109.00 a
		Se1	307.00 ab	305.67 ab	115.00 a	118.00 a
		Se2	304.00 ab	299.67 b	109.33 a	107.67 a
		Se3	300.17 b	300.67 ab	111.33 a	103.67 a
		Se4	306.50 ab	303.33 ab	111.50 a	110.33 a
2022	D1	Se0	292.83 a	301.98 a	94.67 a	114.45 a
		Se1	294.50 a	303.20 a	98.83 a	113.42 a
		Se2	293.17 a	301.33 a	96.67 a	106.55 a
		Se3	294.50 a	300.70 a	94.00 a	106.57 a
		Se4	290.33 a	304.72 a	94.00 a	107.12 a
	D2	Se0	300.25 a	304.78 a	98.50 a	111.32 a
		Se1	298.33 a	303.32 a	100.83 a	107.53 a
		Se2	297.33 ab	305.12 a	98.17 a	92.20 a
		Se3	300.17 a	304.05 a	93.83 a	110.37 a
		Se4	287.67 b	302.73 a	92.33 a	107.88 a
Y			**	NS	**	NS
D			NS	NS	*	NS
T			NS	**	NS	NS
Y×D			NS	NS	NS	NS
Y×T			NS	**	NS	NS
D×T			NS	*	NS	NS
Y×D×T			NS	NS	NS	NS

Description of treatment groups: Se0: Se fertilizer application of 0 g ha^−1^; Se1: Se fertilizer application of 75 g ha^−1^; Se2: Se fertilizer application of 150 g ha^−1^; Se3: Se fertilizer application of 225 g ha^−1^; Se4: Se fertilizer application of 300 g ha^−1^. Different lowercase letters in the same column indicate significant differences among different treatments (*p* < 0.05). NS: not significant. * Significant at the 0.05 probability level. ** Significant at the 0.01 probability level.

**Table 7:** Effect of gradient application of selenium fertilizer on internodal length of dryland maize in 2021 and 2022.

Year	Density	Treatment	Internode Length (cm)					
			3rd Tasseling	4th Tasseling	5th Tasseling	3rd Milk Stage	4th Milk Stage	5th Milk Stage
2021	D1	Se0	13.98 a	17.80 a	19.47 a	13.73 a	18.10 a	20.70 a
		Se1	13.68 a	17.45 a	20.22 a	12.83 a	14.23 a	18.33 a
		Se2	13.02 a	16.38 a	19.87 a	11.97 a	15.07 a	18.00 a
		Se3	14.35 a	18.02 a	20.30 a	14.10 a	17.37 a	21.60 a
		Se4	13.03 a	15.90 a	18.82 a	13.93 a	18.83 a	20.23 a
	D2	Se0	14.33 a	17.38 a	20.62 a	15.97 a	19.50 a	21.53 a
		Se1	14.08 a	17.55 a	20.45 a	13.80 b	17.17 ab	19.97 ab
		Se2	14.95 a	17.23 a	19.93 a	13.10 b	16.97 ab	19.87 ab
		Se3	13.78 a	17.58 a	20.45 a	12.67 b	15.87 b	18.07 bc
		Se4	14.27 a	17.05 a	20.03 a	13.83 b	15.23 b	17.40 c
2022	D1	Se0	9.03 a	12.28 bc	16.70 ab	12.23 a	16.55 a	18.90 a
		Se1	8.88 a	13.20 ab	17.72 a	13.57 a	16.33 a	18.47 a
		Se2	8.77 a	12.08 c	16.53 ab	11.78 a	15.62 a	18.63 a
		Se3	9.48 a	13.73 a	18.22 a	11.42 a	14.90 a	16.00 a
		Se4	8.52 a	11.92 c	15.82 b	11.93 a	15.87 a	18.53 a
	D2	Se0	9.41 a	13.05 a	17.20 a	12.72 a	16.68 a	18.62 a
		Se1	9.78 a	14.53 a	19.32 a	12.23 a	16.82 a	18.47 a
		Se2	9.72 a	13.13 a	17.58 a	12.40 a	16.47 a	18.57 a
		Se3	9.50 a	13.03 a	17.03 a	11.67 a	15.23 a	17.40 a
		Se4	9.37 a	13.23 a	16.63 a	12.35 a	15.67 a	18.07 a
Y			**	**	**	**	*	**
D			*	NS	NS	NS	NS	NS
T			NS	NS	*	*	**	NS
Y×D			NS	NS	NS	NS	NS	NS
Y×T			NS	NS	NS	NS	*	NS
D×T			NS	NS	NS	NS	**	NS
Y×D×T			NS	NS	NS	NS	*	*

Description of treatment groups: Se0: Se fertilizer application of 0 g ha^−1^; Se1: Se fertilizer application of 75 g ha^−1^; Se2: Se fertilizer application of 150 g ha^−1^; Se3: Se fertilizer application of 225 g ha^−1^; Se4: Se fertilizer application of 300 g ha^−1^. Different lowercase letters in the same column indicate significant differences among different treatments (*p* < 0.05). NS: not significant. * Significant at the 0.05 probability level. ** Significant at the 0.01 probability level.

**Table 8:** Effect of gradient application of selenium fertilizer on the internodal diameter of dryland maize in 2021 and 2022.

Year	Density	Treatment	Internode Diameter (mm)					
			3rd Tasseling	4th Tasseling	5th Tasseling	3rd Milk Stage	4th Milk Stage	5th Milk Stage
2021	D1	Se0	28.41 a	27.28 a	27.45 a	23.34 a	22.93 a	22.36 b
		Se1	28.61 a	27.25 a	26.55 a	25.57 a	25.08 a	25.05 ab
		Se2	28.22 a	27.13 a	26.93 a	25.76 a	26.06 a	24.97 ab
		Se3	27.48 a	26.47 a	26.14 a	26.10 a	26.87 a	25.88 a
		Se4	29.84 a	29.20 a	28.49 a	25.23 a	25.38 a	25.98 a
	D2	Se0	26.88 a	26.35 a	26.32 a	22.65 a	23.39 a	22.98 a
		Se1	25.85 a	24.80 a	23.89 a	23.04 a	22.50 a	22.33 a
		Se2	27.67 a	27.03 a	26.37 a	23.72 a	23.29 a	21.87 a
		Se3	26.56 a	25.71 a	24.45 a	23.05 a	22.53 a	22.14 a
		Se4	27.64 a	27.11 a	25.84 a	24.25 a	23.02 a	22.42 a
2022	D1	Se0	24.98 a	27.20 a	27.28 a	30.27 a	27.12 a	22.78 a
		Se1	23.65 a	25.02 bc	24.58 b	28.55 a	25.53 a	22.57 a
		Se2	23.82 a	23.53 c	23.60 b	30.08 a	27.30 a	25.78 a
		Se3	23.70 a	26.35 ab	23.47 b	31.17 a	28.50 a	24.70 a
		Se4	23.07 a	24.95 bc	24.33 b	30.75 a	26.73 a	25.75 a
	D2	Se0	22.63 a	24.85 a	24.85 ab	30.70 a	26.92 a	25.12 a
		Se1	22.51 a	24.05 a	22.10 b	31.43 a	27.26 a	26.79 a
		Se2	24.37 a	26.20 a	25.30 a	31.39 a	29.66 a	25.44 a
		Se3	23.73 a	25.56 a	25.11 ab	31.09 a	28.58 a	23.47 a
		Se4	23.39 a	24.42 a	23.09 ab	30.24 a	27.54 a	24.59 a
Y			**	**	**	**	**	*
D			**	*	**	NS	NS	NS
T			NS	NS	**	NS	NS	NS
Y×D			NS	NS	NS	**	**	**
Y×T			NS	*	NS	NS	NS	NS
D×T			NS	NS	*	NS	NS	NS
Y×D×T			NS	NS	NS	NS	NS	NS

Description of treatment groups: Se0: Se fertilizer application of 0 g ha^−1^; Se1: Se fertilizer application of 75 g ha^−1^; Se2: Se fertilizer application of 150 g ha^−1^; Se3: Se fertilizer application of 225 g ha^−1^; Se4: Se fertilizer application of 300 g ha^−1^. Different lowercase letters in the same column indicate significant differences among different treatments (*p* < 0.05). NS: not significant. * Significant at the 0.05 probability level. ** Significant at the 0.01 probability level.

**Table 9:** Effect of gradient application of selenium fertilizer on stem strength of dryland maize in 2021 and 2022.

Year	Density	Treatment	Stem Strength (N)					
			3rd Tasseling	4th Tasseling	5th Tasseling	3rd Milk Stage	4th Milk Stage	5th Milk Stage
2021	D1	Se0	51.13 b	49.20 b	45.53 c	52.73 a	53.57 a	43.13 a
		Se1	54.97 b	50.20 b	48.10 c	50.53 a	43.13 c	49.53 a
		Se2	70.63 a	65.40 a	62.60 ab	52.10 a	52.97 a	49.30 a
		Se3	69.87 a	67.07 a	66.23 a	48.60 a	51.00 ab	43.67 a
		Se4	52.10 b	53.00 b	53.33 bc	49.60 a	44.33 bc	47.47 a
	D2	Se0	50.80 bc	53.10 a	48.00 a	46.73 a	44.27 a	48.37 a
		Se1	48.70 c	51.50 a	47.97 a	43.40 a	41.27 a	41.60 b
		Se2	58.63 ab	62.73 a	52.27 a	43.30 a	48.00 a	42.13 b
		Se3	63.73 a	60.87 a	56.57 a	44.47 a	42.83 a	38.37 b
		Se4	62.00 a	63.17 a	57.87 a	46.20 a	45.03 a	52.37 a
2022	D1	Se0	57.38 a	50.48 a	44.30 a	76.67 a	66.35 a	59.37 a
		Se1	54.18 a	44.80 a	41.22 a	67.57 a	57.62 b	53.30 a
		Se2	55.70 a	51.25 a	48.40 a	68.07 a	62.25 ab	56.23 a
		Se3	62.65 a	52.58 a	51.63 a	72.85 a	63.15 ab	56.42 a
		Se4	59.83 a	50.65 a	48.68 a	71.07 a	61.93 ab	57.65 a
	D2	Se0	55.30 a	52.05 a	47.10 a	72.25 a	65.53 a	56.35 a
		Se1	57.62 a	52.42 a	49.15 a	69.63 a	63.32 a	55.55 a
		Se2	58.30 a	51.45 a	46.17 a	68.65 a	60.07 a	55.00 a
		Se3	56.02 a	53.40 a	51.48 a	64.55 a	57.15 a	53.48 a
		Se4	56.38 a	57.93 a	52.20 a	65.03 a	63.63 a	57.05 a
Y			NS	**	**	**	**	**
D			NS	NS	NS	**	*	NS
T			**	**	**	NS	**	**
Y×D			NS	NS	NS	NS	*	NS
Y×T			NS	*	NS	NS	NS	NS
D×T			NS	NS	*	NS	*	NS
Y×D×T			*	NS	NS	NS	NS	**

Description of treatment groups: Se0: Se fertilizer application of 0 g ha^−1^; Se1: Se fertilizer application of 75 g ha^−1^; Se2: Se fertilizer application of 150 g ha^−1^; Se3: Se fertilizer application of 225 g ha^−1^; Se4: Se fertilizer application of 300 g ha^−1^. Different lowercase letters in the same column indicate significant differences among different treatments (*p* < 0.05). NS: not significant. * Significant at the 0.05 probability level. ** Significant at the 0.01 probability level.

## Data Availability

Data are contained within the article.
